# Development of a Green-Synthesized WA-CDs@MIL-101 Fluorescent Sensor for Rapid Detection of *Panax notoginseng* Leaf Pathogen Spores

**DOI:** 10.3390/plants14152316

**Published:** 2025-07-26

**Authors:** Chunhao Cao, Wei Sun, Ling Yang, Qiliang Yang

**Affiliations:** 1 Yunnan Key Laboratory of Efficient Utilization and Intelligent Control of Agricultural Water Resources, Kunming University of Science and Technology, Kunming 650500, China; caochunhao93@163.com; 2 Seasonal Arid Region, Water-Soil-Crop System Observation and Research Station of Yunnan Province, Kunming University of Science and Technology, Kunming 650500, China; 3 Faculty of Modern Agricultural Engineering, Kunming University of Science and Technology, Kunming 650500, China; sueiiu@163.com; 4 Yunnan Technology Innovation Center of Phosphorus Resource, Kunming 650600, China; 5 Faculty of Information Engineering and Automation, Kunming University of Science and Technology, Kunming 650500, China

**Keywords:** spore, wax apple, *Panax notoginseng*, fluorescence, leaf disease

## Abstract

The leaf diseases of *Panax notoginseng* (*Panax notoginseng* (Burk) F. H. Chen) are mainly spread by spores. To enable rapid and sensitive detection of spores for early warning of disease spread, we developed a carbon dot-based fluorescent probe encapsulated by MIL-101 using wax apple as a green carbon source (WA-CDs@MIL-101). The WA-CDs@MIL-101 was thoroughly characterized, and the detection conditions were optimized. The interaction mechanism between WA-CDs@MIL-101 and spores was investigated. The fluorescence of WA-CDs@MIL-101 was recovered due to electrostatic adsorption between spores and WA-CDs@MIL-101. Under the optimized detection conditions, the probe exhibited excellent sensing performance, showing a strong linear relationship (*R*^2^ = 0.9978) between spore concentration (0.0025–5.0 mg/L) and fluorescence recovery ratio, with a detection limit of 5.15 μg/L. The WA-CDs@MIL-101 was successfully applied to detect spores on *Panax notoginseng* leaves, achieving satisfactory recoveries (94–102%) with relative standard deviations of 1.3–3.4%. The WA-CDs@MIL-101 shows great promise for detecting spores on *Panax notoginseng* leaves.

## 1. Introduction

*Panax notoginseng* (*Panax notoginseng* (Burk) F. H. Chen), a perennial plant in the Araliaceae family, is extensively cultivated in the Yunnan and Guangxi provinces of China. Its leaves are commonly consumed as functional food ingredients or brewed into health-oriented beverages, owing to their nutritional and bioactive properties [1]. However, the crop is typically grown under warm and humid conditions, which make the leaves particularly vulnerable to foliar diseases. These diseases are primarily transmitted through pathogenic spores on the leaf surface, posing potential threats to both crop quality and food safety [2]. Recent studies have identified several fungal pathogens responsible for leaf diseases in *Panax notoginseng*, including species from the genera *Alternaria* [3], *Fusarium* [4], and *Colletotrichum* [5]. These pathogens are known to cause leaf spot, blight, and anthracnose, significantly reducing photosynthetic capacity, delaying growth, and compromising leaf quality. More importantly, the spores of these fungi are the primary agents of disease transmission, making their early detection essential. Once infection becomes visible, lesions often expand rapidly, resulting in diminished market value and posing potential safety concerns for edible leaf use [6]. Therefore, targeting the spores of these pathogens at the asymptomatic stage is crucial for effective disease management, food quality control, and sustainable cultivation of edible *Panax notoginseng*. Current detection strategies rely largely on macroscopic symptom identification or image recognition, which are reactive and insufficient for early intervention [7,8]. By the time visible symptoms emerge, disease progression is typically advanced, limiting the efficacy of control measures.

To address this, spore-level detection has become a focus for early disease warning. Conventional techniques such as microscopic counting, culture-based methods, and quantitative polymerase chain reaction (qPCR) provide high specificity but suffer from drawbacks—such as labor intensity, long assay times, or poor field portability. Therefore, there is a strong demand for a rapid, sensitive, and field-deployable method for spore detection on leaf surfaces. Fluorescent carbon dots (CDs) [9,10] have emerged as promising nanomaterials in bioassays, offering advantages like high photostability, excellent water solubility, tunable emission, and facile surface modification [11,12,13]. These properties enable strong fluorescence responses and selective recognition of biological targets, including fungal spores. In particular, the use of biomass-derived CDs has gained attention due to their sustainability and environmental friendliness. Wax apple (*Syzygium samarangense*), a tropical fruit widely cultivated in Southeast Asia and Southern China, was selected as a green carbon source for carbon dot synthesis due to its unique compositional and ecological advantages. It is rich in carbohydrates (8.0–13.0% soluble solids), proteins, amino acids, vitamins (especially vitamin C), polyphenols, anthocyanins, and organic acids. These bioactive components not only provide an abundant carbon skeleton but also contribute nitrogen and oxygen atoms for heteroatom doping during synthesis, thereby enhancing the fluorescence performance, surface activity, and stability of the resulting carbon dots. Moreover, natural antioxidants such as polyphenols and anthocyanins promote the formation of conjugated structures, which help improve the fluorescence quantum yield (QY) and tune the emission wavelength. The inherent hydroxyl and carboxyl groups improve water solubility and biocompatibility, which are essential for stable dispersion and biosensing applications. Additionally, wax apple is low-cost, eco-friendly, and renewable, making it a sustainable and economically viable precursor for large-scale, green synthesis of functional nanomaterials. To further enhance the stability and sensitivity of CDs, incorporating them into metal–organic frameworks (MOFs) like MIL-101 has proven effective [14,15,16]. MIL-101, featuring a large surface area and uniform porosity, can encapsulate CDs to prevent aggregation and improve fluorescence consistency. It also enhances interaction with spores via electrostatic forces or hydrogen bonding [17]. The resulting composite—WA-CDs@MIL-101—offers improved dispersion, durability, and detection reproducibility in complex environments.

In this work, we developed a MIL-101-encapsulated carbon dot composite (WA-CDs@MIL-101) synthesized from wax apple juice as a green carbon source. The composite was applied as a fluorescent probe for detecting fungal spores on the surface of *Panax notoginseng* leaves. The specific objectives were (1) to prepare highly fluorescent CDs using wax apple as the green carbon source and to comprehensively characterize their morphology, crystal structure, surface functional groups, chemical bonds, and optical properties; (2) to investigate the sensing mechanism, selectivity, and sensitivity of WA-CDs@MIL-101 for spore detection under optimized conditions; (3) to evaluate the real-sample applicability and anti-interference capability of the probe and validate its accuracy via comparison with qPCR.

## 2. Materials and Methods

### 2.1. Chemicals and Materials

N,N-Dimethylformamide (≥99.8%), acetic acid, terephthalic acid, chromium (III) nitrate nonahydrate (Cr(NO_3_)_3_·9H_2_O), ammonia solution (AR), absolute ethanol (99.5%), and quinine sulfate (98%) were obtained from Macklin Biochemical Technology Co., Ltd. (Shanghai, China). Dialysis membranes were obtained from Union Carbide Corporation (Seadrift, TX, USA), and PES membranes were supplied by Biochrom Biosciences Co., Ltd. (Changde, Hunan, China). All chloride-containing interferents, common sodium salts with anions, glucose, sucrose, ascorbic acid, aspartate, sodium dihydrogen phosphate (NaH_2_PO_4_), disodium hydrogen phosphate (Na_2_HPO_4_), and potassium bromide (KBr, SP, ≥99.9%) were produced by T-Jkemao Chemical Reagents Co., Ltd. (Tianjin, China). Chloroform:isoamyl alcohol (24:1) and isopropanol (analytical grade) were purchased from Sinopharm Chemical Reagent Co., Ltd. (Shanghai, China). RNase-free water was supplied by Thermo Fisher Scientific (Waltham, MA, USA). SYBR® Premix Ex Taq™ II was purchased from Takara Bio Inc. (Shiga, Japan), and primers were synthesized by Sangon Biotech Co., Ltd. (Shanghai, China). All other reagents were of analytical grade and used without further purification. Ultrapure water was used throughout all experiments. Wax apple was sourced from a local market in Kunming, China. Spores were isolated from infected *Panax notoginseng* leaves collected from the cultivation base of the Faculty of Modern Agricultural Engineering, Kunming University of Science and Technology (Jinning, Yunnan, China). Based on prior studies and preliminary microscopic examination, the primary fungal pathogens present in these samples were identified as *Alternaria*, *Fusarium*, and *Colletotrichum* species, which are commonly associated with foliar diseases in *Panax notoginseng* under warm and humid conditions. These fungi are known to produce airborne spores that contribute to rapid disease spread, making early detection critical for effective disease control and food safety assurance. All figures were plotted using Origin 2019 (OriginLab Corporation, Northampton, MA, USA), and all calculations were performed using Microsoft Excel 2016 (Microsoft Corporation, Redmond, WA, USA).

### 2.2. Synthesis of WA-CDs@MIL-101 Composite

**Figure 1:**
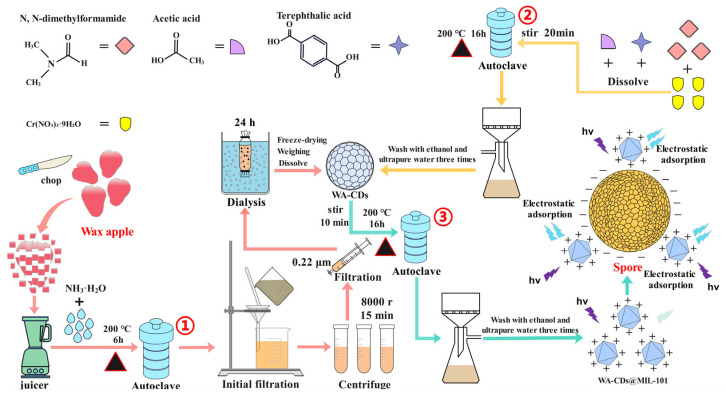
Preparation process and detection principle of WA-CDs@MIL-101 with wax apple (*Syzygium samarangense*) as the green carbon source.

The WA-CDs@MIL-101 composite was prepared (Figure 1) by first synthesizing carbon dots (WA-CDs) using wax apple as the carbon source, followed by their incorporation into the MIL-101 (MOF). To prepare WA-CDs [18], fresh wax apples were cut into small pieces (5.0 g) and juiced using a juicer. The obtained juice was mixed with 2.0 mL of ammonia solution (NH_3_·H_2_O) as a modifier. The mixture was then transferred into a Teflon-lined stainless-steel autoclave and subjected to hydrothermal treatment at 200 °C for 6 h to facilitate the formation of WA-CDs. After the reaction, the product was initially filtered using filter paper to remove large particulates, followed by centrifugation at 8000 rpm for 15 min to further purify the WA-CD solution. The resulting supernatant was filtered through a 0.22 μm PES membrane, and the filtrate was dialyzed (1000 Da) to remove small molecular impurities. Finally, the purified WA-CDs were obtained by freeze-drying.

For the synthesis of MIL-101, 10.0 mL of N,N-dimethylformamide (DMF), 0.5 mL of acetic acid, 1 mmol of terephthalic acid, and 2 mmol of Cr(NO_3_)_3_·9H_2_O were mixed and stirred magnetically for 20 min to ensure complete dissolution and homogenization. The mixture was then transferred into the autoclave and heated at 200 °C for 16 h to promote the formation of MIL-101. After cooling, the product was washed three times with ethanol and ultrapure water to remove unreacted precursors and by-products. To prepare the WA-CDs@MIL-101 composite, the obtained WA-CDs were dissolved and added into the MIL-101 precursor solution, followed by stirring for 10 min to promote uniform dispersion of WA-CDs. The resulting mixture was then subjected to another hydrothermal treatment at 200 °C for 16 h, allowing the WA-CDs to be successfully incorporated into the MIL-101 framework. The final product was washed three times with ethanol and ultrapure water to remove unbound WA-CDs and other residual impurities. The resulting composite was denoted as WA-CDs@MIL-101. When spores were mixed with WA-CDs@MIL-101, electrostatic adsorption occurred between them, causing a reconfiguration of the surface electronic states or energy levels of WA-CDs@MIL-101. These changes disrupted the quenching pathway induced by Cr^3+^, eventually leading to fluorescence recovery.

### 2.3. Characterization and Fluorescence Detection

The particle size distributions of WA-CDs and WA-CDs@MIL-101 were analyzed using dynamic light scattering (DLS) with a nanolaser particle size analyzer (ZS90, Malvern, UK). In addition, this instrument was employed to determine the zeta potentials of WA-CDs@MIL-101, fungal spores, and their mixture. Crystalline properties of WA-CDs@MIL-101 and MIL-101 were investigated using an X-ray diffractometer (XRD, Miniflex 600, Rigaku, Tokyo, Japan), with the scan range set from 5° to 60°. Transmission electron microscope (TEM) image of WA-CDs@MIL-101 was obtained using a JEM-2100 Plus microscope (JEOL, Tokyo, Japan) to further examine the microstructure of the composite. Scanning electron microscope (SEM) image and energy dispersive X-ray spectroscopy (EDX) of WA-CDs@MIL-101 were obtained using a Sigma 300 microscope (Zeiss, Jena, Germany). Functional groups present on WA-CDs@MIL-101 and MIL-101 were identified using Fourier transform infrared spectroscopy (FTIR, Nicolet iS20, Thermo Fisher Scientific, Waltham, MA, USA). X-ray photoelectron spectroscopy (XPS) of WA-CDs@MIL-101 was carried out with a K-Alpha system (Thermo Scientific, Waltham, MA, USA), and all binding energies were calibrated with reference to the C 1s peak at 284.8 eV. The fluorescence properties of WA-CDs and WA-CDs@MIL-101 were characterized using a fluorescence spectrophotometer (F-4600, Hitachi, Tokyo, Japan). The ultraviolet-visible (UV-vis) absorption spectra of WA-CDs@MIL-101 and spores + WA-CDs@MIL-101 were recorded using a UV-vis spectrophotometer (T9CS, Purkinje, Beijing, China). DNA concentration and purity were measured using a NanoDrop 2000 spectrophotometer (Thermo Scientific, Waltham, MA, USA). qPCR was conducted on a CFX96 Touch Real-Time PCR Detection System (Bio-Rad, Hercules, CA, USA). The detection of fungal spores was performed by monitoring fluorescence recovery under optimized conditions (pH 7.0, 25 °C, 20 min). Experimental optimization, interference and QY studies are provided in SI (Sections S1.1–S1.4).

### 2.4. Calibration and Real Sample Analysis

Standard curves were established using isolated spores and qPCR validation. Real sample detection was performed using spiked *Panax notoginseng* leaf extracts. Details are shown in SI (Sections S1.5–S1.6).

## 3. Results and Discussion

### 3.1. Characterization

**Figure 2:**
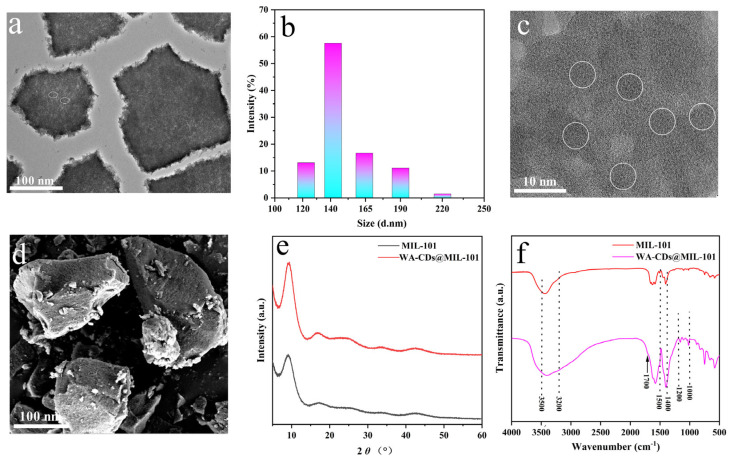
(**a**) Transmission electron microscopy image, (**b**) Particle size distribution, (**c**) Transmission electron microscopy magnified image (WA-CDs are identified using circles), and (**d**) Scanning electron microscopy image of WA-CDs@MIL-101. (**e**) X-ray diffraction spectra, (**f**) Fourier transform infrared spectra of WA-CDs and WA-CDs@MIL-101.

The TEM image (Figure 2a) shows that WA-CDs@MIL-101 exhibits a polyhedral MIL-101 structure with particle sizes mainly between 100–200 nm and uniform dispersion. Numerous dark, spherical nanoparticles (<10 nm) are observed on or within the MIL-101 matrix, corresponding to WA-CDs [19]. Their even distribution and absence of aggregation suggest successful loading via physical adsorption or coordination. Slightly blurred lattice edges in some areas imply partial doping or encapsulation. These features confirm effective integration of WA-CDs into MIL-101 and suggest altered surface or optical properties that may influence fluorescence behavior.

As shown in Figure 2b, WA-CDs@MIL-101 exhibits a unimodal particle size distribution centered at ~140 nm, with good dispersibility and size uniformity. Minor peaks at 120–220 nm suggest minimal aggregation. Figure 2c (enlarged TEM) further confirms that WA-CDs are uniformly embedded or anchored within the MIL-101 framework without notable aggregation. Figure 2d (SEM) shows a rough surface with small particles likely corresponding to WA-CDs, indicating successful surface loading. EDX analysis (Figure S1) reveals dominant elements C (47.81%), O (34.43%), and Cr (17.76%), consistent with the hybrid’s organic (WA-CDs) and inorganic (MIL-101) nature. The high atomic percentage of carbon (61.48%) confirms effective WA-CD incorporation. Absence of detectable nitrogen is likely due to EDX’s limited sensitivity to light elements.

The XRD spectra (Figure 2e) show that MIL-101 retains sharp diffraction peaks, indicating good crystallinity. In contrast, WA-CDs@MIL-101 exhibits reduced peak intensity and a more diffuse background, suggesting decreased crystallinity due to WA-CD incorporation [20]. While the characteristic peak around 10° remains, indicating partial structural retention, the flattening of the profile above 30° reflects the presence of amorphous CDs and partial framework disorder.

The structural variations of MIL-101 and WA-CDs@MIL-101 were further investigated by FTIR spectra (Figure 2f). FTIR spectra further corroborate the discovery of XRD. Both MIL-101 and WA-CDs@MIL-101 exhibit typical MOF vibrational features. For instance, stretching vibrations of aromatic C=C bonds appear in the 1500–1400 cm^−1^ region [21], while the peak near 1700 cm^−1^ is associated with the stretching vibration of carboxylic C=O groups [22]. In the WA-CDs@MIL-101 spectrum, additional absorption bands appear in the 3200–3500 cm^−1^ region, [23] which can be attributed to O–H or N–H stretching vibrations from the WA-CDs, indicating their successful incorporation. Moreover, the enhanced absorption bands in the range of 1200–1000 cm^−1^ may be associated with C–O–C or C–N stretching vibrations [24], further confirming the presence of WA-CDs. In summary, the combined XRD and FTIR analyses confirm the successful loading of WA-CDs onto MIL-101. WA-CDs not only influence the crystallinity of MIL-101 but also introduce additional surface functional groups. These modifications may enhance the adsorption capability of the composite toward target molecules and provide more active sites for subsequent optical applications.

**Figure 3:**
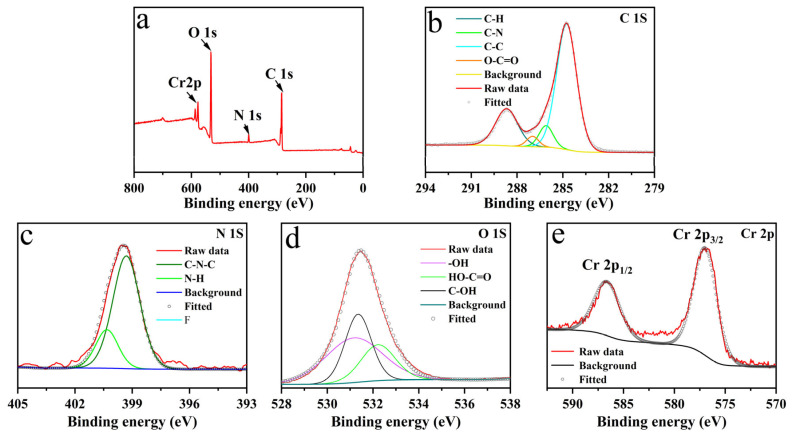
Survey X-ray photoelectron spectrum (**a**), high-resolution X-ray photoelectron spectrum of C1S (**b**), N1S (**c**), O1S (**d**), and Cr2p (**e**) of WA-CDs@MIL-101.

The chemical composition and interaction mechanism of WA-CDs@MIL-101 were further investigated through the combination of XPS analyses (Figure 3). The XPS survey spectrum (Figure 3a) reveals that the composite mainly consists of carbon (C), oxygen (O), nitrogen (N), and chromium (Cr) elements. The presence of Cr confirms the retention of the MIL-101 metal framework, while the increased relative contents of C and O suggest the successful introduction of WA-CDs, which are rich in oxygen-containing functional groups. High-resolution XPS spectra provide further insights. In the C 1S spectrum (Figure 3b), peak deconvolution reveals contributions from C–C (284.8 eV) [25], C–N (285.7 eV) [26], and O–C=O (288.6 eV) [27] bonds. The appearance of the O–C=O peak is likely related to the carboxyl groups from WA-CDs, supporting their successful incorporation. The N 1S spectrum (Figure 3c) exhibits peaks at 399.6 eV and 401.2 eV, which can be assigned to C–N–C and N–H bonds, respectively [28]. This implies that nitrogen-containing species, such as pyridinic or amide groups, have been introduced by WA-CDs. The O 1S spectrum (Figure 3d) shows distinct peaks at 531.8 eV, 532.5 eV, and 533.2 eV, corresponding to C–OH, –OH, and HO–C=O groups [29], respectively, indicating the abundance of oxygen-containing functional groups on the WA-CDs surface. These functionalities may enhance the hydrophilicity and surface reactivity of the composite. The Cr 2p spectrum (Figure 3e) displays two main peaks corresponding to Cr 2p_3/2_ and Cr 2p_1/2_, further confirming the structural integrity of the MIL-101 framework after WA-CD loading. In summary, the XPS results, in conjunction with the XRD and FTIR data, provide solid evidence for the successful modification of MIL-101 by WA-CDs. This modification not only alters the crystallinity of MIL-101 but also introduces a variety of O- and N-containing functional groups. These changes are expected to enhance the adsorption properties of the material and offer additional active sites for potential applications.

**Figure 4:**
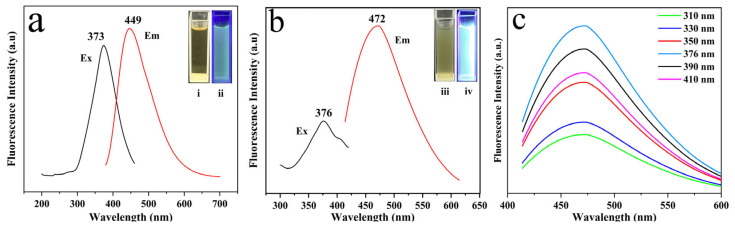
(**a**) Fluorescence excitation, emission spectra of WA-CDs (inset: photographs of WA-CDs under unexcited (i) and 373 nm light source (ii) excitation). (**b**) Fluorescence excitation, emission spectra of spores + WA-CDs@MIL-101 (inset: photographs of spores + WA-CDs@MIL-101 under unexcited (iii) and 376 nm light source (iv) excitation). (**c**) Fluorescence emission spectra of spores + WA-CDs@MIL-101 under excitation light sources ranging from 310 to 410 nm.

Figure 4a shows that WA-CDs emit strong blue fluorescence under 373 nm excitation (i and ii), with an emission peak at 449 nm and a large Stokes shift of 76 nm. Figure 4b reveals that after binding with spores, the excitation and emission peaks of WA-CDs@MIL-101 shift to 376 nm and 472 nm, respectively, indicating red shift likely caused by interactions such as electrostatic adsorption or π–π stacking. The composite exhibits bright blue-green fluorescence under UV (iii and iv), confirming responsiveness. Figure 4c shows excitation-dependent emission behavior, peaking at 376 nm, suggesting multiple emission centers. The stable peak position across excitation wavelengths highlights strong binding and excellent detection performance.

**Figure 5:**
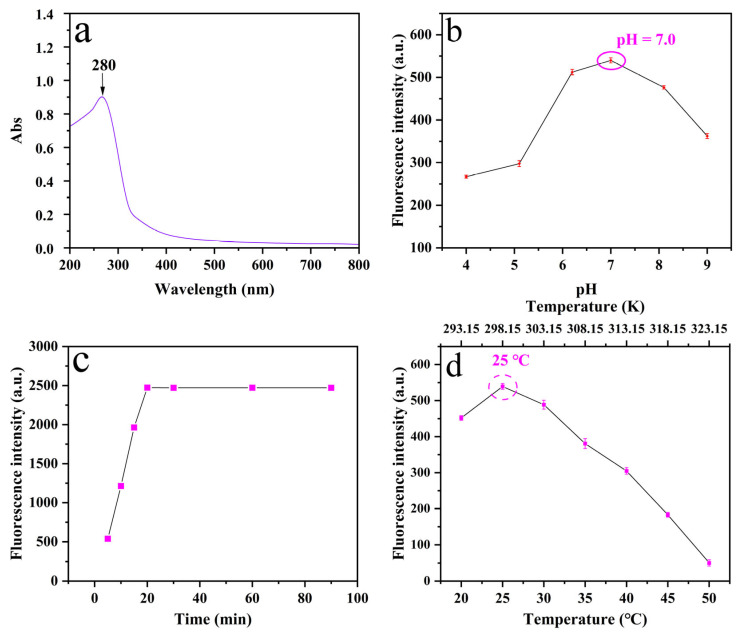
(**a**) Ultraviolet–visible absorption spectrum of WA-CDs@MIL-101, (**b**) Effect of pH on the fluorescence intensity of WA-CDs@MIL-101, (**c**) Reaction equilibrium time of spores with WA-CDs@MIL-101, (**d**) Effect of temperature on the fluorescence intensity.

As shown in the UV–vis absorption spectrum (Figure 5a), WA-CDs@MIL-101 exhibits prominent absorption characteristics in the range of 200–800 nm. A distinct absorption peak is observed around 280 nm [30], which is typically attributed to π→π* electronic transitions, likely originating from aromatic ring structures or conjugated C=C bonds within the WA-CDs. Following this peak, the absorption intensity decreases rapidly and levels off beyond 300 nm, indicating minimal light absorption in the visible range (400–800 nm), which is consistent with the non-colored appearance of the material under visible light. This absorption behavior further confirms the successful incorporation of WA-CDs into the MIL-101 framework while retaining the characteristic π-conjugated structure of WA-CDs. In conjunction with the results from XRD, FTIR, and XPS analyses, the UV–vis spectrum provides additional evidence for the successful hybridization of WA-CDs with MIL-101 and the resulting changes in the composite’s optical properties. Moreover, the π→π* transition absorption [31] observed near 280 nm may also be associated with the formation of new electronic structures following the composite formation, suggesting possible electronic interactions between WA-CDs and the MIL-101 matrix. The involvement of Cr^3+^ d-orbitals from MIL-101 could also influence the absorption characteristics, implying that the chromium centers not only serve as structural nodes in the MOF framework but also play a key role in maintaining the stability and functionality of the WA-CDs@MIL-101 composite through their oxidation and coordination states.

### 3.2. Optimization of Experimental Conditions

To optimize fluorescence performance, the effect of pH on WA-CDs@MIL-101 was evaluated (Figure 5b). Fluorescence intensity peaked at pH 7.0, while acidic and alkaline conditions led to quenching. This is likely due to protonation or deprotonation of surface groups (e.g., –COOH, –OH) on WA-CDs and possible structural disruption of MIL-101 under extreme pH, which impair fluorescence emission. Moreover, the altered hydrogen bonding network in alkaline media may facilitate the approach of quenchers such as hydroxide ions (OH^−^), further contributing to fluorescence quenching, which is consistent with previous studies [32]. Based on these findings, pH 7.0 was selected as the optimal condition for subsequent experiments.

In the incubation time optimization experiment of the WA-CDs@MIL-101 composite, seven different time points (5, 10, 15, 20, 30, 60, and 90 min) were set to systematically investigate the fluorescence response under fixed concentration and pH conditions. The experimental results (Figure 5c) showed that the fluorescence intensity gradually increased within the first 20 min, with a particularly significant rise observed between 5 and 20 min. After 20 min, the fluorescence intensity tended to stabilize, indicating that the reaction system had reached adsorption or reaction equilibrium around 20 min. This trend primarily reflects the kinetic characteristics of the interaction between spores and WA-CDs@MIL-101. In the early stage of incubation, spores rapidly bind to the surface of the composite via electrostatic interactions, coordination, or hydrogen bonding, resulting in a sharp increase in fluorescence signal. As time progresses, the available binding sites on WA-CDs@MIL-101 are gradually occupied by spores, leading to saturation of adsorption and stabilization of the fluorescence signal, suggesting that the system has reached kinetic equilibrium. At this point, the structure of the composite material tends to be stable, and the fluorescence emission efficiency reaches its maximum. During prolonged incubation beyond 20 min, the fluorescence signal no longer increases significantly and even shows a slight decline in some samples, which may be attributed to the aggregation of spores on the MIL-101 surface, energy transfer, or self-quenching phenomena. It is also possible that slight microstructural changes occurred in WA-CDs@MIL-101 during extended incubation, thereby affecting its ability to support and immobilize the spores. Therefore, based on the experimental data, 20 min is considered the optimal incubation time, as it ensures sufficient interaction while maintaining the structural stability of WA-CDs@MIL-101 and maximizing the fluorescence response, providing valuable guidance for the construction of an efficient and rapid detection system.

In the process of using WA-CDs@MIL-101 composites for the detection of *Panax notoginseng* leaf disease spores, seven incubation temperature conditions (20, 25, 30, 35, 40, 45, and 50 °C) were set to explore the optimal temperature for the reaction between the spores and the fluorescent probe, while keeping other variables (pH and incubation time) constant. The fluorescence recovery response of the system under different temperatures was investigated. As shown in Figure 5d, the fluorescence intensity reached its maximum at 25 °C, and then gradually decreased with increasing temperature, indicating that room temperature (25 °C) is the most favorable condition for fluorescence response during the incubation process. This phenomenon may be closely related to the activity states of spore surface-active substances (such as proteins, polysaccharides, or metabolic products) at different temperatures.

At 25 °C, the spores exhibit moderate metabolic activity and structural stability, allowing the active molecules released from their surfaces (e.g., amines, thiol compounds, etc.)—which contribute to fluorescence recovery—to sufficiently interact with the WA-CDs@MIL-101 composites and restore part of the fluorescence signal quenched by metal ions. However, when the temperature drops below 25 °C, the fluorescence intensity decreases due to the reduced activity of WA-CDs@MIL-101. When the temperature increases to 30 °C or above, the physiological state of the spores may change, and the release rate of active components becomes either too fast or unstable, thereby reducing the efficiency of fluorescence signal recovery. Meanwhile, elevated temperatures may enhance the thermal motion of the metal centers (Cr^3+^) within the MIL-101 framework, aggravating fluorescence quenching and further weakening the signal.

In addition, higher temperatures might affect the membrane permeability of the spores, consequently altering the diffusion efficiency of their metabolites and weakening the interaction between these metabolites and the fluorescent centers on the WA-CDs@MIL-101 surface, thereby failing to effectively trigger the fluorescence recovery mechanism. Therefore, the experimental results clearly indicate that 25 °C is the optimal incubation temperature for achieving the best fluorescence response between the spores and WA-CDs@MIL-101, which can greatly enhance detection sensitivity and response efficiency, and provide important experimental support for the subsequent development of rapid spore identification and disease early-warning platforms.

### 3.3. Selectivity and Anti-Interference Ability

**Figure 6:**
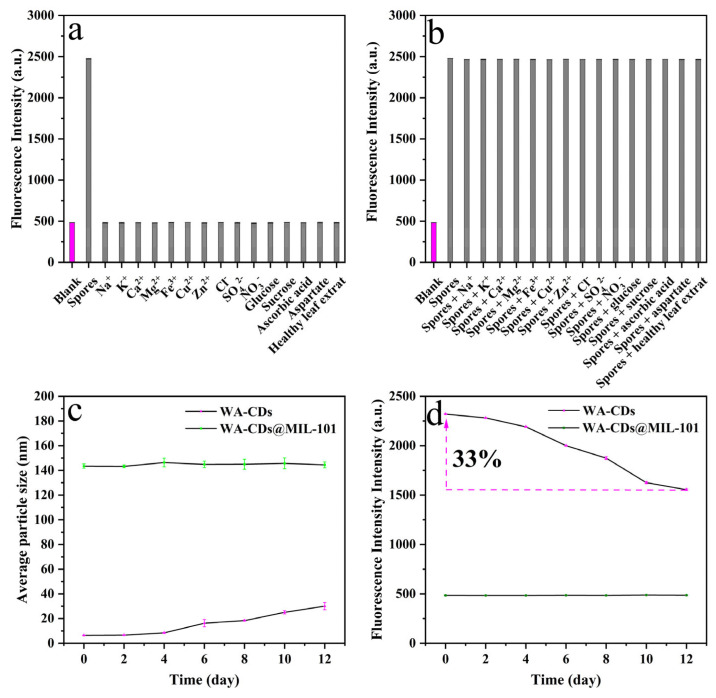
Test results of selectivity and anti-interference ability of WA-CDs@MIL-101 when spores and interfering substances do not coexist (**a**) and coexist (**b**) in the detection system. (**c**) Average particle size and (**d**) fluorescence intensity changes of WA-CDs and WA-CDs@MIL-101 within 0-12 days.

As shown in Figure 6a,b, whether the interfering substances and spores coexist in the detection system or not, the interference of the interfering substances on the detection results was relatively small, indicating that these substances had no significant enhancement or quenching effect on the fluorescence of WA-CDs@MIL-101. Notably, although metal ions such as Fe^3+^ and Cu^2+^ are often reported to induce quenching or signal fluctuations in other fluorescent sensing systems, no interference was observed in this system, suggesting that WA-CDs@MIL-101 exhibits high tolerance to these ions and possesses excellent selectivity and stability. Moreover, the extract from healthy leaves also failed to restore the fluorescence signal, showing almost no difference from the blank group, further confirming the probe’s recognition specificity toward pathogenic spores. In contrast, when a standard solution of diseased spores (5.0 mg/L) was added, the fluorescence signal recovered significantly, far exceeding the responses triggered by other interfering substances, displaying a typical “off–on” fluorescence response pattern. This response may originate from specific interactions—such as hydrogen bonding, electrostatic attraction, or defect passivation—between active groups on the spore surface and WA-CDs@MIL-101, thereby modulating the fluorescence emission process.

In summary, the results of the selectivity experiment demonstrate that the WA-CDs@MIL-101 fluorescent probe exhibits good recognition specificity and anti-interference capability toward *Panax notoginseng* leaf disease spores, providing theoretical support and technical assurance for its practical application in complex plant samples.

### 3.4. Verification of the Inhibitory Effect of MIL-101 on the Aggregation Behavior of WA-CDs

As shown in Figure 6c, the average particle size of WA-CDs was approximately 6.3 nm on day 0 and gradually increased to around 30 nm over 12 days, indicating time-dependent aggregation. In contrast, WA-CDs@MIL-101 maintained a stable particle size of ~140 nm throughout the same period, demonstrating that the MIL-101 framework effectively inhibited WA-CDs aggregation. Figure 6d further shows that the fluorescence intensity of WA-CDs decreased to ~67% of its initial value over 12 days, consistent with aggregation-induced quenching. However, WA-CDs@MIL-101 exhibited negligible fluorescence loss, confirming that MIL-101 not only suppresses aggregation but also preserves fluorescence by limiting energy transfer between particles.

### 3.5. Standard Curve

Figure S2a,b illustrates the linear relationship between fluorescence recovery response and the fluorescence spectra of WA-CDs@MIL-101 and varying concentrations of *Panax notoginseng* leaf disease spores. The spore concentration was plotted on the *x*-axis, while the fluorescence recovery ratio (*F/F*_0_) was plotted on the *y*-axis, where *F* represents the fluorescence intensity after spore addition and *F*_0_ denotes the initial fluorescence intensity without spores. The results showed that the *F/F*_0_ values gradually increased with rising spore concentrations, demonstrating a clear concentration-dependent fluorescence enhancement trend. Within the concentration range of 0.0025–5.0 mg/L, a good linear relationship was observed between *F/F*_0_ (*y*) and the spore concentration (*x*) (mg/L), as described by Equation (1).

*F/F*_0_ = *y* = 0.805*x* + 1.059 (1)

The *R*^2^ was 0.9978, indicating excellent quantitative detection capability within this range. This behavior may be attributed to certain metabolites in the pathogenic spores—such as amino acids, phenolic compounds, or proteins—that possess reducing properties or can specifically interact with the defect sites on the surface of WA-CDs. These substances effectively alleviate the fluorescence-quenching effect of MIL-101 on WA-CDs, thereby enabling partial recovery of the fluorescence signal. The slope of 0.805 obtained from the linear regression model reflects the sensor’s high response sensitivity. Specifically, it denotes the quantitative change in fluorescence intensity ratio per unit concentration of spores (mg/L), highlighting the sensor’s capability to detect subtle variations in spore levels with high precision. This reflects superior detection performance and application potential. In conclusion, the WA-CDs@MIL-101 system can be effectively applied for the quantitative detection of pathogenic spores, offering excellent sensitivity, a wide linear range, and good reproducibility, thus laying a solid foundation for the development of early spore recognition and field-based disease warning systems. The limit of detection (LOD) was calculated by Equation (2).

LOD = 3*σ*/*K* (2)

where *σ* represents the standard deviation of repeated measurements of 11 blank samples, and *K* is the slope of the calibration curve. In this study, the slope was determined to be *K* = 0.805. Based on this calculation, the LOD of the proposed method was found to be 5.15 μg/L. This low LOD indicates that the WA-CDs@MIL-101 system is capable of detecting *Panax notoginseng* leaf disease spores at trace levels, demonstrating high sensitivity and suitability for early warning applications in *Panax notoginseng* disease monitoring.

Compared with previously reported analytical methods, the WA-CDs@MIL-101 sensor developed in this work exhibits significant advantages in terms of sensitivity and detection range. As summarized in Table 1, most conventional methods focus on small-molecule analytes such as antibiotics (e.g., tetracyclines, enrofloxacin), dyes (e.g., methylene blue), and organic pollutants (e.g., dichlorophenol), with LODs typically ranging from 0.04 to 98.0 mg/L. In contrast, the WA-CDs@MIL-101 system achieves an exceptionally low LOD of 0.00515 mg/L for spore detection, making it the most sensitive among the compared methods. Moreover, its linear detection range of 0.0025–5.0 mg/L covers both trace and moderate concentrations, offering a broader and more practical application window than many existing sensors.

### 3.6. Detection Mechanism

To further elucidate the underlying mechanism of spore-induced fluorescence recovery in the WA-CDs@MIL-101 system, the changes in zeta potential before and after incubation with *Panax notoginseng* leaf disease spores were investigated. As shown in Figure S2c, the untreated WA-CDs@MIL-101 exhibited a distinctly positive surface charge (+25.83 mV), which is presumably attributed to the coordinated metal ions in WA-CDs@MIL-101 and the presence of residual cationic species. In contrast, the spores of *Panax notoginseng* leaf disease typically exhibit a slightly negative surface charge (−12.47 mV), owing to the abundance of proteins, polysaccharides, and other metabolic byproducts on their surface. After incubation with the spores, the zeta potential of WA-CDs@MIL-101 significantly decreased to +2.6 mV, suggesting that anionic groups on the spore surface (e.g., carboxyl, hydroxyl, phosphate groups) were adsorbed onto the composite material, thereby altering its surface charge distribution. This phenomenon of charge neutralization or reversal is likely to interfere with the Cr^3+^-mediated fluorescence-quenching process within WA-CDs@MIL-101, thereby reducing non-radiative relaxation and restoring the fluorescence emission of WA-CDs. These results preliminarily confirm that the fluorescence recovery is closely related to electrostatic interactions induced by the spores, implying that the spores can modulate the local surface environment of the material via electrostatic regulation, leading to optical signal changes.

Furthermore, UV–vis absorption spectroscopy was employed to study the optical absorption behavior of WA-CDs@MIL-101 before and after exposure to the spores. As illustrated in Figure S2d, the pristine WA-CDs@MIL-101 exhibited characteristic π-π* absorption peaks (around 230–280 nm) and some n-π* transitions (320–360 nm), which correspond to the conjugated structure and surface functional groups of the WA-CDs. Upon reaction with the *Panax notoginseng* spores, a significant enhancement and red shift in the absorption band between 300 and 400 nm was observed. This indicates the formation of new energy levels or intermediate states on the material surface, likely caused by the adsorption of spore-derived proteins or carbohydrates, which, in turn, may facilitate new energy transfer pathways or suppress quenching mechanisms. This spectral shift was consistent with the observed fluorescence recovery, further supporting the hypothesis that the spores modulate the optical behavior of the composite material.

In summary, the change in zeta potential demonstrates significant electrostatic adsorption between the spores and the WA-CDs@MIL-101 surface, while the UV–vis spectral variations indicate a possible reconfiguration of surface electronic states or energy levels. These changes likely disrupt the metal-induced quenching pathway, ultimately leading to fluorescence recovery. Together, these findings provide strong theoretical and experimental evidence supporting the detection mechanism based on spore-triggered fluorescence restoration.

### 3.7. Real Sample Analysis

To evaluate the detection reliability of WA-CDs@MIL-101 in real complex samples, this study employed *Panax notoginseng* leaf extract as the matrix and performed spiked recovery experiments to assess the accuracy of the WA-CDs@MIL-101-based method. According to the established linear range of the calibration curve (0.0025–5.0 mg/L), three concentration levels—low (0.5 mg/L), medium (2.0 mg/L), and high (4.0 mg/L)—were selected. Equal volumes of spore standard solutions were spiked into blank leaf extracts and tested under optimized experimental conditions. The standard curve for quantifying spore concentration using the qPCR method is presented in Figure S3. A strong linear relationship was observed between the Ct values and the logarithm of known spore DNA concentrations, with an *R*^2^ of 0.9984, indicating excellent linearity and analytical reliability. This high degree of linearity confirms the robustness of the qPCR assay and its suitability as a reference method for accurate quantification of *Panax notoginseng* leaf pathogen spores. Consequently, the qPCR method was employed as the standard molecular tool to validate the performance, accuracy, and practical applicability of the WA-CDs@MIL-101 fluorescence-based sensor in this study.

The results (Table 2) showed that the spiked recoveries at the three levels were 94, 99, and 102%, respectively, with RSDs all below 5%. These findings indicate that the method exhibits excellent accuracy, reproducibility, and stability in real sample matrices. Moreover, the results further demonstrate the strong applicability of the WA-CDs@MIL-101 fluorescent probe for quantitative detection of plant disease spores, showing great potential for early warning applications in the complex environment of *Panax notoginseng* leaves.

To validate the reliability of the fluorescence method, samples from the same batch were simultaneously analyzed using both the WA-CDs@MIL-101 sensor and the conventional qPCR method for comparison. At spiking levels of 0.5, 2.0, and 4.0 mg/L, the spore concentrations determined by qPCR were approximately 0.5, 1.9, and 3.9 mg/L, respectively, with recoveries ranging from 96% to 97% and RSDs below 3.5%, indicating high accuracy and repeatability. In comparison, the WA-CDs@MIL-101 fluorescent sensor yielded spore concentrations of approximately 0.5, 2.0, and 4.1 mg/L at the same spiking levels, corresponding to recoveries of 94–102% with RSDs also within 3.5%. The results obtained from both methods were in good agreement (*p* > 0.05), confirming the consistency and reliability of the fluorescence-based method.

In conclusion, the WA-CDs@MIL-101 fluorescence sensing method not only maintains high sensitivity but also offers excellent accuracy and strong application equivalence to standard qPCR assays. This method provides an effective, rapid, low-cost, and portable detection strategy for on-site spore monitoring and early warning in plant disease management.

## 4. Conclusions

In this study, a fluorescent probe (WA-CDs@MIL-101) was developed using wax apple-derived carbon dots and MIL-101 as a protective matrix for detecting spores on *Panax notoginseng* leaves. The composite (~140 nm) was characterized by TEM, XRD, FTIR, and XPS, confirming its morphology, amorphous structure, and functionalized surface. It exhibited strong fluorescence (λ_ex/λ_em = 376/472 nm) with a large Stokes shift of 76 nm. Optimal detection conditions (pH 7.0, 25 °C, 20 min) yielded a linear fluorescence response (*R*^2^ = 0.9978) over 0.0025–5.0 mg/L with a low LOD of 5.15 μg/L. The fluorescence recovery was attributed to electrostatic interactions between spores and the probe. MIL-101 improved the dispersion and stability of WA-CDs. The probe showed high selectivity and anti-interference ability, with recoveries of 94–102% and RSDs of 1.3–3.4% in real leaf samples, comparable to qPCR (*p* > 0.05). This study highlights the significance of early detection of pathogenic fungal spores (e.g., *Alternaria*, *Fusarium*, *Colletotrichum*) associated with *Panax notoginseng* leaf diseases. Rapid and sensitive detection at the spore level enables timely disease intervention, effectively reducing the impact on crop quality, yield, and food safety. These findings underscore the critical role of the *Panax notoginseng*–pathogen pathosystem and demonstrate the practical value of the WA-CDs@MIL-101 probe in real-world agricultural monitoring.

This work provides a green, sensitive, and practical tool for early detection of fungal spores. Future efforts will focus on enhancing probe design and integrating intelligent analysis tools (e.g., machine learning) for field applications.

## Figures and Tables

**Table 1:** Assay performance of different analytical methods in the available reports.

Analytes	Technique	Linear Range (mg/L)	Limit of Detection (mg/L)	Reference
Organic pollutants	BES ^a^	0.2–1	0.1	[33]
Dichlorophenol	FEAB ^b^	0.5–2	0.5	[34]
Tetracycline	FCDMP ^c^	4–20	0.2	[35]
Methylene blue	HOSN ^d^	40–100	98.04	[36]
Enrofloxacin	GCDC ^e^	1–50	0.04	[37]
Tetracyclines	DUECD ^f^	0.5–40	0.07061	[38]
Spores	WA-CDs@MIL-101	0.0025–5	0.00515	This work

^a^ Bio-electrochemical sensor (BES). ^b^ Freeze-dried electrochemically active bacteria (FEAB). ^c^ Fluorescent carbon dots by flow-assisted melt polymerization (FCDMP). ^d^ Hybrid graphene oxide-immobilized silver nanocomposite (HOSN). ^e^ Green fluorescent carbon dots from chitosan (GCDC). ^f^ Deep ultraviolet emissive carbon dots (DUECD).

**Table 2:** Results of the spore spiked assay in real samples (*n* = 3).

Sample	Method	Spiked (mg/L)	Detected (mg/L)	Recovery (%)	RSD (%)
*Panax notoginseng* leaf	WA-CDs@MIL-101	0	/	/	/
		0.50	0.47	94	2.5
		2.00	1.98	99	1.3
		4.00	4.08	102	3.4
	qPCR	0	/	/	/
		0.50	0.48	96	3.3
		2.00	1.93	97	2.2
		4.00	3.88	97	1.9

Note: *p* > 0.05, / means undetected.

## Data Availability

Data can be obtained from authors via email.

## Supplementary Materials

The following supporting information can be downloaded at: https://www.mdpi.com/article/10.3390/plants14152316/s1, Figure S1. Energy dispersive X-Ray spectroscopy of WA-CDs@MIL-101. Figure S2. (a) Fluorescence recovery ratio of WA-CDs@MIL-101 linearly with spore concentration, and (b) Fluorescence stacking spectra after different concentrations of spores were added to WA-CDs@MIL-101. (c) Zeta potentials of WA-CDs@MIL-101, spores and WA-CDs@MIL-101 + spores. (d) Ultraviolet-visible absorption spectra of WA-CDs@MIL-101 and WA-CDs@MIL-101 + spores. Figure S3. Standard curve of quantitative Polymerase Chain Reaction (qPCR) for quantifying spore concentration based on cycle threshold (Ct) values. References [39,40,41] are cited in the supplementary materials.

## Abbreviations

The following abbreviations are used in this manuscript:

CDs	Carbon Dots
WA-CDs	Wax Apple-Derived Carbon Dots
MIL-101	Materials of Institute Lavoisier-101 (a type of metal–organic framework)
WA-CDs@MIL-101	Composite of WA-CDs encapsulated in MIL-101
QY	Quantum Yield
MOFs	Metal–Organic Frameworks
TEM	Transmission Electron Microscopy
SEM	Scanning Electron Microscopy
EDX	Energy-Dispersive X-ray Spectroscopy
DLS	Dynamic Light Scattering
XRD	X-ray Diffraction
FTIR	Fourier Transform Infrared Spectroscopy
XPS	X-ray Photoelectron Spectroscopy
UV–vis	Ultraviolet–Visible Spectroscopy
qPCR	Quantitative Polymerase Chain Reaction
LOD	Limit of Detection
RSD	Relative Standard Deviation
SI	Supporting Information
FCDMP	Fluorescent Carbon Dots by Flow-Assisted Melt Polymerization
BES	Bio-Electrochemical Sensor
FEAB	Freeze-Dried Electrochemically Active Bacteria
HOSN	Hybrid Graphene Oxide-Immobilized Silver Nanocomposite
GCDC	Green Fluorescent Carbon Dots from Chitosan
DUECD	Deep Ultraviolet Emissive Carbon Dots

## References

[1] Zhang X., Zhang B., Zhang C., Sun G., Sun X. (2020). Effect of *Panax notoginseng* saponins and major anti-obesity components on weight loss. Front. Pharmacol..

[2] Yang K., Wang H.L., Ye C., Wang Z.H., Ye K.H., Zhang S., Huang H.P., Wei Z.X., Zhu S.S., Zhu Y.Y. (2022). Infection characteristics and physical prevention strategy of *Panax notoginseng* round spot disease caused by mycocentrospora acerina. Plant Dis..

[3] Li X., Li S., Qiu B., Zhang Y., Cui X., Ge F., Liu D. (2020). Thaumatin-like protein genes of *Panax notoginseng* confers resistance to *Alternaria* panax. Physiol. Mol. Plant Pathol..

[4] Zhou Y., Liu Y., Li S., Yang Q. (2024). The combination of biochar and bacillus subtilis biological agent reduced the relative abundance of pathogenic bacteria in the rhizosphere soil of *Panax notoginseng*. Microorganisms.

[5] Chen C.-J., Li Q.-Q., Zeng Z.-Y., Duan S.-S., Wang W., Xu F.-R., Cheng Y.-X., Dong X. (2020). Efficacy and mechanism of Mentha haplocalyx and Schizonepeta tenuifolia essential oils on the inhibition of *Panax notoginseng* pathogens. Ind. Crops Prod..

[6] Hu Y., Zhang H., Lu Y., Ao D., Liang Z., Zhao M., Yang S., Tang Q. (2024). Microencapsulation of total saponins from stem and leaf of *Panax notoginseng* by freeze and spray drying: Process optimization, physicochemical properties, structure, antioxidant activity, and stability. J. Food Sci..

[7] Wang R., Zhang X., Yang Q., Lei L., Liang J., Yang L. (2024). Enhancing *Panax notoginseng* leaf disease classification with Inception-SSNet and image generation via improved diffusion model. Agronomy.

[8] Wang Z., Yang L., Wang R., Lei L., Ding H., Yang Q. (2024). WE-DeepLabV3+: A lightweight segmentation model for *Panax notoginseng* leaf diseases. Comput. Electron. Agric.

[9] Yi K., Zhang X., Zhang L. (2020). Eu^3+^@metal–organic frameworks encapsulating carbon dots as ratiometric fluorescent probes for rapid recognition of anthrax spore biomarker. Sci. Total Environ..

[10] Ma M., Pan J., Wang J., Jing Y., Fu Y., Shen Y., Wang D., Wang C., Li J. (2025). Cu^2+^-cross-linked tannic acid carbon dot nanoparticles for mold inhibition. ACS Appl. Nano Mater..

[11] Gao X., Zhang H., Liu L., Jia M., Li X., Li J. (2024). Nano-biosensor based on manganese dioxide nanosheets and carbon dots for dual-mode determination of Staphylococcus aureus. Food Chem..

[12] Ren Y., Fan Z. (2023). Synthesis of fluorescent probe based on molecularly imprinted polymers on nitrogen-doped carbon dots for determination of tobramycin in milk. Food Chem..

[13] Wang M., Zeng L., Chen H., Hu S., Huang C., Zhen S., Zhan L. (2025). Antibacterial properties of folic acid-based hydrogel loaded with CeCDs and its potential application in food preservation. Food Chem..

[14] Wang Z., Yan L., Song Z., Zhang J., Yang Y., Liu X. (2025). Revealing the in-situ growth mechanism of carbon dots confined in ZIF-8 as multicolor fluorescent material with high photothermal stability. J. Colloid Interface Sci..

[15] Tan P., Chen Y., Chang H., Liu T., Wang J., Lu Z., Sun M., Su G., Wang Y., Wang H.D. (2024). Deep learning assisted logic gates for real-time identification of natural tetracycline antibiotics. Food Chem..

[16] Wang Y., Wei X., Su Y., Xu R., Song D., Ding L., Chen Y. (2024). Highly sensitive fluoroprobe for detecting Sudan dyes in paprika utilizing carbon dot-embedded zeolitic imidazolate framework-8. Food Chem..

[17] Li W., Yu X., Tang Y., Li Z., Shah S.J., Liu Y., Zhao H., Song M., Li J., Wang G. (2023). Confined construction of CDs@MIL-101 as reusable and turn-on fluorescence sensor for highly sensitive water detection based on adsorption-solvent synergistic mechanism. Sens. Actuators B Chem..

[18] He Q., Zhuang S., Yu Y., Li H., Liu Y. (2021). Ratiometric dual-emission of Rhodamine-B grafted carbon dots for full-range solvent components detection. Anal. Chim. Acta.

[19] Li C., Li N., Yang L., Liu L., Zhang D. (2024). Synthesis of fluorescent carbon dots by B/P doping and application for Co^2+^ and methylene blue detection. Spectrochim. Acta A Mol. Biomol. Spectrosc..

[20] Zhang Y., Hu K., Yuan B., Zhu X., Chen X., Huang K. (2023). Gasoline residue-prepared carbon dots embedded metal–organic frameworks for selective and sensitive detection of sulfide ions. Microchem. J..

[21] Li Y., Chi C., Zhao Y., Jiang G., Wu J., Song J. (2025). Formaldehyde detection based on tannin carbon dots. Chem. Eng. Sci..

[22] Zhao Z., Jing Y., Shen Y., Liu Y., Wang J., Ma M., Pan J., Wang D., Wang C., Li J. (2024). Silicon-doped carbon dots crosslinked carboxymethyl cellulose gel: Detection and adsorption of Fe^3+^. Gels.

[23] McEnroe A., Brunt E., Mosleh N., Yu J., Hailstone R., Sun X. (2023). Bright, green fluorescent carbon dots for sensitive and selective detection of ferrous ions. Talanta Open.

[24] Yang D., Ma C., Chen G., Li L., Hu A., Huang A., Zhou Y., Cai Z., Yang T., Gao H. (2025). Investigation of the application and mechanism of nitrogen and phosphorus co-doped carbon dots for mercury ion detection. J. Fluoresc..

[25] Yang D., Lin J., Ying W., Wen P., Zhang J., Chen Z. (2025). Xylooligosaccharides, monosaccharides, and pH-sensitive carbon dots production from Toona sinensis branches using organic acid hydrolysis and hydrothermal treatment. Int. J. Biol. Macromol..

[26] Zhu B., Zhang K., Wei S., Li S., Peng D., Nie J., Duan J., Wu D., Chang X. (2025). Converting waste chrysanthemi flos residues into high-value fluorescent carbon dots for rapid and selective detection of mercury (II) ions in aqueous environments. Ind. Crops Prod..

[27] Ren C., Zhang M., Zheng N., Liu B., Tang J., Tang J., Zhang F., Chen G. (2025). Green synthesis of carbon dots and their application as fluorescent probes for rutin detection. Spectrochim. Acta A Mol. Biomol. Spectrosc..

[28] Zhao C.-Y., Tseng W.-B., Hung K.-H., Tseng W.-L. (2025). Ultrasensitive detection of tetracycline using the disruption of crosslink-enhanced emission and inner-filter effect-induced phosphorescence quenching of carbonized polymer dots. Biosens. Bioelectron..

[29] Miao C., Shi X., Li Z., Zhang X., Wang X., Yang D., Wang Q. (2025). Norfloxacin-derived carbon dots with rich electron pyrrolic nitrogen for copper corrosion inhibition and antibacterial functions. Langmuir.

[30] Maria S.A.P., Lima G.M., André R., Matiuzzi C.M., Leonardo D.B., Pequeno O.H. (2025). Antibacterial activity of ciprofloxacin-based carbon dot@silver nanoparticle composites. ACS Omega.

[31] Atchudan R., Karuppasamy B.D., Perumal S., Gangadaran P., Sundramoorthy A.K., Manoj D., Rajendran R.L., Ahn B.-C., Ahamed M., Lee S.W. (2025). Sustainable-biomass-derived multifunctional carbon dots as fluorescent probes for multi-purpose advanced imaging, migration and security solutions. Surf. Interfaces.

[32] Jalili R., Irani-nezhad M.H., Khataee A., Joo S.W. (2021). A ratiometric fluorescent probe based on carbon dots and gold nanocluster encapsulated metal–organic framework for detection of cephalexin residues in milk. Spectrochim. Acta A Mol. Biomol. Spectrosc..

[33] Zang Y., Zhao T., Xie B., Feng Y., Yi Y., Liu H. (2021). A bio-electrochemical sensor based on suspended Shewanella oneidensis MR-1 for the sensitive assessment of water biotoxicity. Sens. Actuators B Chem..

[34] Zang Y., Cao B., Zhao H., Xie B., Ge Y., Yi Y., Liu H. (2023). On-site determination of water toxicity based on freeze-dried electrochemically active bacteria. Sci. Total Environ..

[35] Liu Z., Ni L., Chi J., Qin Y., Shi Z., Wang Y., Wei H., Feng L., Sun C. (2024). Preparation of fluorescent carbon dots by flow-assisted melt polymerization for tetracycline detection in medical wastewater. Chin. J. Chromatogr..

[36] Dat N.M., Quan T.H., Nguyet D.M., Anh T.N.M., Thinh D.B., Diep T.C., Huy L.A., Tai L.T., Hai N.D., Khang P.T. (2021). Hybrid graphene oxide-immobilized silver nanocomposite with optimal fabrication route and multifunctional application. Appl. Surf. Sci..

[37] Xu J., Qi Q., Sun L., Guo X., Zhang H., Zhao X. (2022). Green fluorescent carbon dots from chitosan as selective and sensitive “off-on” probes for nitrite and “on-off-on” probes for enrofloxacin detection. J. Alloys Compd..

[38] Yin W., Gu J., Zhu T., Gao H., Ma C., Zhu C., Li L., Yang Z., Chen G. (2022). Use of deep ultraviolet emissive carbon dots as a novel optical sensor for the detection of tetracyclines in milk. IEEE Sens. J..

[39] Wu M., Liu T., Yin C., Jiang X., Sun Q., Gao L., Niu N., Chen L., Gang H. (2023). Portable smartphone-assisted RGB-dependent ratiometric sensing platform for the detection of tetrachloro-p-benzoquinone in river samples. Microchem. J..

[40] Zhang Y., Cheng S., Zhang Y. (2023). Green fluorescent carbon dots for sensing of quercetin and pH and cell imaging. Luminescence.

[41] Hernández-Rodríguez M.A., Afonso M.M., Palenzuela J.A., Martín I.R., Soler-Carracedo K. (2018). Carbon dots as temperature nanosensors in the physiological range. J. Lumin..

