# Supplementary material for: Development of a Green-Synthesized WA-CDs@MIL-101 Fluorescent Sensor for Rapid Detection of Panax notoginseng Leaf Pathogen Spores
Source: Plants (Basel). 2025 Jul 26;14(15):2316. doi: 10.3390/plants14152316 (PMC12348358; doi:10.3390/plants14152316)
Supplement: Supplementary file 1 [file plants-14-02316-s001.zip › plants-3762726-supplementary.pdf]

Supporting Information

Development of a green-synthesized WA-CDs@MIL-101 fluorescent sensor for rapid detection of *Panax notoginseng* leaf pathogen spores

Chunhao Cao [1,2,3,4], Wei Sun [3], Ling Yang [5,*], Qiliang Yang [1,2,3,4,*]

[1] *Yunnan Provincial Key Laboratory of High-efficiency Water Use and Green Production of Characteristic Crops in Universities, Kunming University of Science and Technology, Kunming 650500, PR China*

[2] *Yunnan Provincial Field Scientific Observation and Research Station on Water-Soil-Crop System in Seasonal Arid Region, Kunming University of Science and Technology, Kunming 650500, PR China*

[3] *Faculty of Modern Agricultural Engineering, Kunming University of Science and Technology, Kunming 650500, PR China*

[4] *Yunnan Technology Innovation Center of Phosphorus Resource, Kunming 650600, PR China*

[5] *Faculty of Information Engineering and Automation, Kunming University of Science and Technology, Kunming 650500, PR China*

[*] Corresponding author: Ling Yang, Ph.D. E-mail: yangling@kust.edu.cn; Qiliang Yang, Ph.D. E-mail: yangqilianglovena@163.com.

S1 Materials and Methods (Detailed)

1.1. Optimization of experimental conditions

To optimize the detection performance of WA-CDs@MIL-101 for spores present on the surface of *Panax notoginseng* leaves, several critical parameters were systematically investigated, including incubation time,[1] pH,[2] and temperature,[3] in order to achieve maximum sensitivity and selectivity. First, various incubation durations (5, 10, 15, 20, 30, 60, and 90 minutes) were tested by mixing the spore suspension with the WA-CDs@MIL-101 composite, and changes in fluorescence intensity were recorded to determine the optimal reaction time required to reach signal saturation. Second, the influence of pH was examined by adjusting the reaction environment to pH values of 4.0, 5.1, 6.2, 7.0, 8.1, and 9.0, using phosphate-buffered saline (PBS) solutions to maintain pH stability. The interactions between WA-CDs@MIL-101 and spores were evaluated under each condition to identify the pH level yielding the strongest fluorescence response. Additionally, the impact of temperature on detection efficiency was assessed by performing experiments at 20, 25, 30, 35, 40, 45, and 50 °C. These studies aimed to elucidate the effects of thermal conditions on the binding stability between WA-CDs@MIL-101 and the spores, as well as on the resulting fluorescence intensity. All experiments were conducted in triplicate to ensure reproducibility. Response curves plotting each variable against fluorescence intensity were constructed to quantify the influence of each parameter on the sensing system. Based on the combined experimental outcomes, optimal detection conditions were determined.

1.2. Verification of the inhibitory effect of MIL-101 on the aggregation behavior of WA-CDs

To evaluate the inhibitory effect of MIL-101 on the potential spontaneous aggregation behavior of WA-CDs in aqueous solution, two sets of samples were

prepared: one consisting of free WA-CDs solution, and the other comprising a dispersion of the WA-CDs@MIL-101 composite. Both samples were prepared at the same concentration and stored under identical conditions, kept in the dark at room temperature without disturbance. Samples were collected at specific time intervals over a 12-day period for the following analyses: (1) DLS was used to measure the particle size distribution of both systems, in order to assess any significant changes in size over time. (2) Fluorescence intensity variations were monitored using a fluorescence spectrophotometer. The excitation wavelengths of the fluorescence spectrophotometer were set to 373 (WA-CDs) and 376 (WA-CDs@MIL-101) nm respectively, and the emission wavelengths were set to 449 (WA-CDs) and 472 (WA-CDs@MIL-101) nm respectively.

1.3. Selectivity and anti-interference ability

To verify the specificity of the constructed WA-CDs@MIL-101 fluorescent probe for recognizing disease-related spores on *Panax notoginseng* leaves in complex sample matrices, a series of selectivity experiments were systematically conducted. These experiments aimed to evaluate the impact of common interfering substances, including inorganic ions, small organic molecules, and healthy leaf extracts, on the fluorescence response of the probe, thereby assessing its anti-interference capability and recognition specificity. Under optimized conditions (pH 7.0, incubation temperature 25 °C, reaction time 20 min), various potential interferents were individually introduced into the WA-CDs@MIL-101 system. These included typical cations (Na^+, K^+, Ca^{2+}, Mg^{2+}, Fe^{3+}, Cu^{2+}), anions (Cl^-, SO_4^{2-}, NO_3^-), small organic compounds (glucose, sucrose, ascorbic acid, aspartate), and extracts from healthy *Panax notoginseng* leaves. The concentration of each interferent was set at 5.0 mg/L, equivalent to the maximum concentration of the target spores used in detection, in order to ensure a consistent basis for comparison.

Subsequently, the fluorescence intensities of each substance before and after addition were recorded respectively, including the situations of coexistence or non-coexistence in the detection system.

1.4. QY

To evaluate the fluorescence performance of the synthesized WA-CDs@MIL-101, the QY was determined using a relative method. Quinine sulfate in 0.1 M H_2SO_4 solution (QY = 54%) was employed as the fluorescence standard. Under identical excitation wavelength (376 nm), the fluorescence emission intensity and absorbance of both the standard and the sample were measured, ensuring that the absorbance values of both were below 0.1 to minimize the inner filter effect. The QY was calculated according to equation (1):

$$Q = Q_s\left(\frac{I_x}{I_s}\right)\left(\frac{A_s}{A_x}\right)\left(\frac{n_x^2}{n_s^2}\right) \qquad (1)$$

Where Q is the quantum yield, I is the integrated fluorescence intensity, A is the absorbance, and n is the refractive index of the solvent. The subscripts "*x*" and "*s*" refer to the sample and the standard, respectively. The results showed that the QY of WA-CDs@MIL-101 was 12.2%, while the QY of the WA-CDs@MIL-101 mixed with spore suspension increased to 22.6%, which was consistent with the observation that the presence of spores recovered the fluorescence intensity of WA-CDs@MIL-101.

1.5. Standard curve

To obtain pathogenic spores from *Panax notoginseng* leaf diseases for fluorescence response experiments, a laboratory-based self-isolation method was employed for the induction, cultivation, and separation of spores from naturally infected leaves. The procedure was conducted as follows: First, *Panax notoginseng* leaves exhibiting visible disease symptoms were collected from the field, with priority given to those showing

typical signs of fungal infections such as downy mildew or gray mold—characterized by grayish-white or purplish-brown mold layers on the abaxial leaf surface or cottony lesions on the adaxial side. The symptomatic leaf tissues were cut into approximately 1 × 1 cm^2 sections. These sections were quickly surface-sterilized in 70% ethanol for 5 seconds, followed by three rinses with sterile distilled water to eliminate surface contaminants. The sterilized leaf segments were then placed in sterile Petri dishes lined with moistened filter paper, ensuring that the paper remained humid but not waterlogged. The dishes were incubated in a constant-temperature chamber at 25 °C under high humidity conditions (relative humidity > 95%) for 48 hours to promote the natural germination or sporulation of the pathogens on the surface of the lesions.

After incubation, 2 mL of sterile water was gently added dropwise onto the surface of the diseased lesions using a sterile pipette. The surface of the lesion was then gently scraped with sterile tweezers to dislodge the spores, and the area was repeatedly rinsed to ensure maximum collection. The resulting spore-containing solution was collected into sterile centrifuge tubes. To remove leaf tissue debris, the suspension was filtered through a double layer of sterile gauze, followed by centrifugation at 3000 rpm for 5 minutes. The supernatant was discarded, and the pellet was resuspended in a pre-determined volume of PBS (pH 7.0) to obtain spore suspensions of various concentrations for subsequent reaction experiments with WA-CDs@MIL-101. Throughout the entire procedure, aseptic techniques were strictly maintained. All instruments were sterilized with 75% ethanol to prevent microbial contamination. The final spore concentration was expressed in mg/L, based on the dry weight of the collected spore pellet resuspended in a defined volume of PBS buffer.

To establish a calibration curve for detecting spores on the surface of *Panax notoginseng* leaves using WA-CDs@MIL-101, a series of spore suspensions with

known concentrations were prepared. Under optimized detection conditions, fluorescence intensities were measured to determine the quantitative relationship between fluorescence response and spore concentration. Initially, a 5 mg/L spore suspension was serially diluted to generate a concentration gradient. Each diluted suspension (0.5 mL) was then mixed with a fixed volume of the WA-CDs@MIL-101 solution (1.0 mL) at optimal pH (7.0), incubated at 25 °C for 20 minutes, and analyzed using a fluorescence spectrophotometer to record fluorescence intensities at different spore concentrations. To eliminate background interference, a blank control group containing only WA-CDs@MIL-101 (without spores) was included. After data collection, a calibration curve was plotted with spore concentration on the x-axis and the fluorescence intensity ratio (F/F_0) on the y-axis. Linear regression analysis was performed to determine the linear equation and correlation coefficient (R^2). To ensure the accuracy and reproducibility of the results, each concentration was tested in triplicate, and the relative standard deviation (RSD) was calculated. Finally, the established standard curve was applied for the quantitative analysis of spores in real samples. The spore concentrations in unknown samples were determined by measuring fluorescence intensities and interpolating from the standard curve, providing a reliable method for rapid and quantitative detection of spore contamination on *Panax notoginseng* leaves. All solutions were stored in amber glass bottles to prevent photodegradation.

1.6. Real sample analysis

To further evaluate the feasibility and accuracy of the developed WA-CDs@MIL-101 fluorescent sensor for the detection of pathogenic spores in real samples, spiked recovery experiments were conducted. Healthy, disease-free leaves of *Panax notoginseng* were first collected and cut into small pieces. Leaf surface extracts were

obtained by soaking and shaking (25 minutes) the samples in sterile saline solution (0.9% NaCl). The resulting extract was then filtered through a 0.22 μm membrane to remove particulate matter and potential interferents, yielding the sample solution for testing. To simulate different levels of spore contamination, standard solutions of *Panax notoginseng* pathogenic spores at low, medium, and high concentrations (corresponding to 10%, 50%, and 80% of the established linear range) were spiked into the leaf extract. Each concentration level was tested in triplicate and recorded as the spiked group.

The spiked samples were incubated with the WA-CDs@MIL-101 fluorescent probe under optimized conditions (pH = 7.0, 25 °C, 20 min). Fluorescence intensities were then measured using a fluorescence spectrophotometer at the optimal excitation and emission wavelengths ($\lambda\_ex = 376$ nm, $\lambda\_em = 472$ nm). The spore concentrations in the spiked samples were calculated based on the previously established standard calibration curve. Recovery rates were determined by comparing the detected concentrations with the known spiked concentrations. The RSD of the three replicates was calculated to assess the accuracy and reproducibility of the method.

To validate the reliability and practical applicability of the WA-CDs@MIL-101 probe for detecting pathogenic spores in *Panax notoginseng* leaves, qPCR was used as a standard molecular reference method. Total DNA was extracted from infected leaves using the cetyltrimethylammonium bromide (CTAB) method. Specifically, 0.5 g of infected leaf tissue was ground in liquid nitrogen, mixed with CTAB extraction buffer, and incubated at 65 °C for 30 minutes. DNA was then extracted using chloroform-isoamyl alcohol, precipitated with isopropanol, washed with 70% ethanol, and finally dissolved in RNase-free water. Specific primers targeting the conserved internal transcribed spacer (ITS) region of fungal spores were designed for qPCR amplification. A standard curve was established using known concentrations of spore DNA (0.0025-

5.0 mg/L), with the cycle threshold (Ct) values logarithmically fitted against spore concentration. A strong linear correlation ($R^2 > 0.99$) was obtained, enabling accurate quantification of spore concentrations in unknown samples.

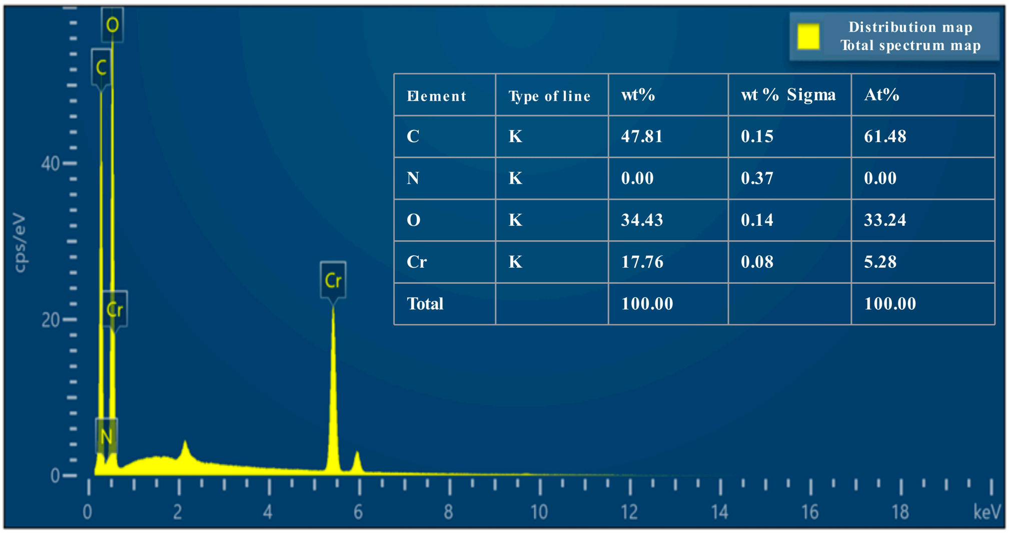

Element	Type of line	wt%	wt % Sigma	At%
C	K	47.81	0.15	61.48
N	K	0.00	0.37	0.00
O	K	34.43	0.14	33.24
Cr	K	17.76	0.08	5.28
Total		100.00		100.00

Figure S1. Energy dispersive X-Ray spectroscopy of WA-CDs@MIL-101.

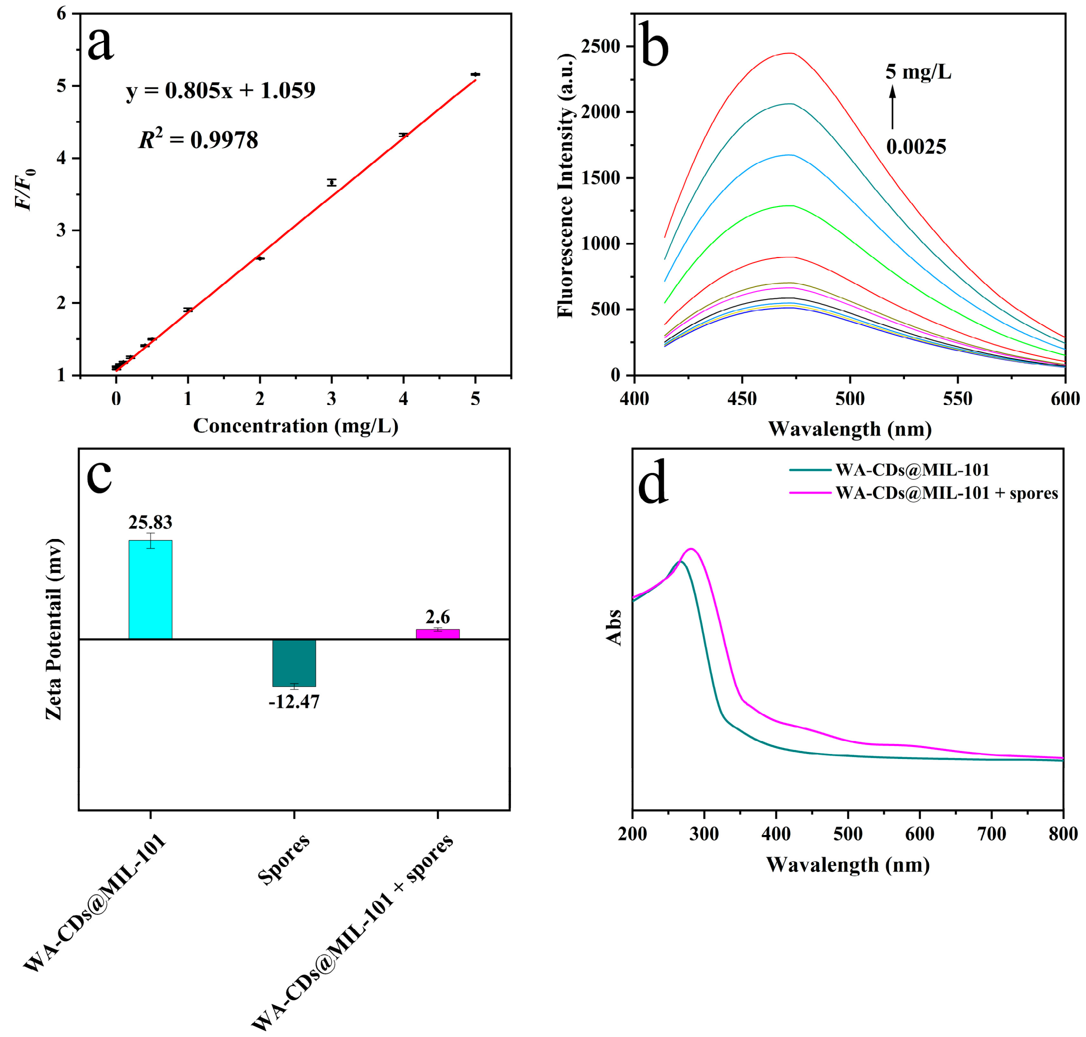

Figure S2. (a) Fluorescence recovery ratio of WA-CDs@MIL-101 linearly with spore concentration, and (b) Fluorescence stacking spectra after different concentrations of spores were added to WA-CDs@MIL-101. (c) Zeta potentials of WA-CDs@MIL-101, spores and WA-CDs@MIL-101 + spores. (d) Ultraviolet-visible absorption spectra of WA-CDs@MIL-101 and WA-CDs@MIL-101 + spores.

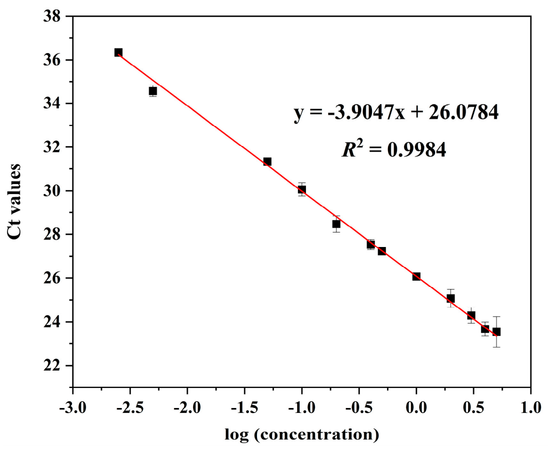

Figure S3. Standard curve of quantitative Polymerase Chain Reaction (qPCR) for quantifying spore concentration based on cycle threshold (Ct) values.

References

(1) Wu, M., Liu, T., Yin, C., Jiang, X., Sun, Q., Gao, L., Niu, N., Chen, L., & Gang, H. Portable smartphone-assisted RGB-dependent ratiometric sensing platform for the detection of tetrachloro-p-benzoquinone in river samples. *Microchem. J.,* **2023**. *190*, 108686.

(2) Zhang, Y., Cheng, S., & Zhang, Y. Green fluorescent carbon dots for sensing of quercetin and pH and cell imaging. *Luminescence,* **2023**. *39*(2), e4638.

(3) Hernández-Rodríguez, M. A., Afonso, M. M., Palenzuela, J. A., Martín, I. R., & Soler-Carracedo, K. Carbon dots as temperature nanosensors in the physiological range. *J. Lumin.,* **2018**. *196*, 313-315.
